# Forced unbinding of GPR17 ligands from wild type and R255I mutant receptor models through a computational approach

**DOI:** 10.1186/1472-6807-10-8

**Published:** 2010-03-16

**Authors:** Chiara Parravicini, Maria P Abbracchio, Piercarlo Fantucci, Graziella Ranghino

**Affiliations:** 1 Department of Pharmacological Sciences, University of Milano, via Balzaretti 9, 20133, Milano, Italy; 2 Department of Biotechnology and Biosciences, University of Milano-Bicocca, Piazza della Scienza 2, 20126 Milano, Italy; 3 Delos S.r.l., via Lurani 12, 20091, Bresso, Italy

## Abstract

**Background:**

GPR17 is a hybrid G-protein-coupled receptor (GPCR) activated by two unrelated ligand families, extracellular nucleotides and cysteinyl-leukotrienes (cysteinyl-LTs), and involved in brain damage and repair. Its exploitment as a target for novel neuro-reparative strategies depends on the elucidation of the molecular determinants driving binding of purinergic and leukotrienic ligands. Here, we applied docking and molecular dynamics simulations (MD) to analyse the binding and the forced unbinding of two GPR17 ligands (the endogenous purinergic agonist UDP and the leukotriene receptor antagonist pranlukast from both the wild-type (WT) receptor and a mutant model, where a basic residue hypothesized to be crucial for nucleotide binding had been mutated (R255I) to Ile.

**Results:**

MD suggested that GPR17 nucleotide binding pocket is enclosed between the helical bundle and extracellular loop (EL) 2. The driving interaction involves R255 and the UDP phosphate moiety. To support this hypothesis, steered MD experiments showed that the energy required to unbind UDP is higher for the WT receptor than for R255I. Three potential binding sites for pranlukast where instead found and analysed. In one of its preferential docking conformations, pranlukast tetrazole group is close to R255 and phenyl rings are placed into a subpocket highly conserved among GPCRs. Pulling forces developed to break polar and aromatic interactions of pranlukast were comparable. No differences between the WT receptor and the R255I receptor were found for the unbinding of pranlukast.

**Conclusions:**

These data thus suggest that, in contrast to which has been hypothesized for nucleotides, the lack of the R255 residue doesn't affect the binding of pranlukast a crucial role for R255 in binding of nucleotides to GPR17. Aromatic interactions are instead likely to play a predominant role in the recognition of pranlukast, suggesting that two different binding subsites are present on GPR17.

## Background

Extracellular adenine and uracil nucleotides (e.g., ATP, ADP, UTP, UDP and sugar nucleotides) are signaling molecules involved in several patho physiological phenomena, from short-term signaling (neurotransmission, mechanosensory transduction, secretion and vasodilatation) to long-term functions (proliferation, differentiation, survival and death, development and post-injury repair) [1]. Conversely, cysteinyl-leukotrienes (cysteinyl-LTs) are inflammatory lipid mediators derived from arachidonic acid through the 5-lypoxigenase (5-LO) pathway, and are implicated in bronchial asthma, stroke and cardiovascular diseases [2]. Despite the fact that nucleotides and cysteinyl-LTs originate from totally independent metabolic pathways, several data suggest important functional interactions between two families of signaling molecules and their receptors. To date, eight distinct nucleotide G-protein-coupled receptors (GPCRs), the P2Y receptors have been identified (P2Y_1;2;4;6;11;12;13;14_) and classified in two distinct phylogenetic subgroups: the first subgroup includes the P2Y_1;2;4;6;11 _subtypes, whereas P2Y_12_, P2Y_13 _and P2Y_14 _belong to the second subgroup [3]. Only two cysteinyl-LTs responding GPCRs (the CysLT1 and CysLT2 receptors) are instead currently recognized. However, certain reported actions of cysteinyl-LTs are not readily explained by interaction with either CysLT1 or CysLT2, raising the possibility of the existence of additional CysLT receptors [4-7]. There exists a functional cross-talk between the P2Y and CysLT receptor families, since both nucleotides and cysteinyl-LTs massively accumulate at sites of inflammation and both types of receptors are co-expressed in the same peripheral inflammatory cells. This evidence shows a cross-regulated response typical of the chemoattractant systems [8]. Along this line, in rat brain microglial cells, both nucleotides and cysteinyl-LTs, that are co-released as a consequence of the activation of P2Y_1 _and CysLT receptors, contribute to neuroinflammation and neurodegeneration [9]. Nucleotides can also regulate, via heterologous desensitization, CysLT1 receptor activity [8] and, in parallel, the CysLT1 receptor antagonists pranlukast and montelukast can functionally influence P2Y receptor signaling pathways in human monocyte/macrophage-like cells [10]. In addition, P2Y_12 _was found to be promiscuously activated by both nucleotides and LTE4 [11], further underlying the close relationship between the two families. Both P2Y and CysLT receptors share the typical seven-transmembrane spanning topology of GPCRs. Besides their heterogeneity in function and tissue distribution, P2Y and CysLT receptors share a phylogenetic relationship, given that both families, together with GPR17 and other related receptors, belong to the so called "purine receptor cluster" of GPCRs [12]. This cluster also includes several "orphan" receptors responding to yet-unidentified endogenous ligands. Among these, the orphan receptor GPR17 appeared to us as a possible common ancestral progenitor that originated the two above receptor families. On this basis, we recently cloned the human, rat and mouse GPR17 and demonstrated that they all respond to both nucleotides and cysteinyl-LTs [13,14].

Thus, GPR17 is a hybrid receptor linking the P2Y and the CysLT receptor families. Besides endogenous ligands, synthetic compounds typical of the two above receptor families are also active at GPR17. Specifically, it has been shown that GPR17 can be activated in vitro by uracil nucleotides (UDP and UDP-sugars) and by cysteinyl-LTs (LTC_4_, LTD_4 _and LTE_4_). GPR17 activation can be contrasted by treatment with two well known P2Y antagonists, MRS2179 (2'-deoxy-N6-methyladenosine 3',5'-biphosphate) and cangrelor (N(6)-(2-methyl-thioethyl)-2-(3,3,3-trifluoropropylthio)-*β*, γ-dichloromethylene-ATP), and also by the already marketed CysLT receptor antagonists pranlukast (N-[4-oxo-2-(2H-tetrazol-5-yl) chromen-7-yl]-4-(4-phenylbutoxy) benzamide) and montelukast (2-[1-[[(1R)-1-[3-[2-(7-chloroquinolin-2-yl) ethenyl] phenyl]-3-[2-(2-hydroxypropan-2-yl) phenyl] propyl] sulfanylmethyl]cyclopropyl] acetic acid). Furthermore, in a model of focal rodent brain ischemia, its *in vivo *early knock down with either pharmacological or specific antisense strategies, reduces the progression of cerebral ischemic damage, highlighting GPR17 as novel therapeutic target for ischemia [13]. Since at present this disease still remains without a specific pharmacological treatment, molecules active as GPR17 inhibitors may represent a new class of promising anti-ischemic agents. On the other hand, more recent data has shown that GPR17 indeed has a dual and spatiotemporal-dependent role in the development and post-injury repair of damage in the brain and in spinal cord. While at very early times after injury, GPR17 seems to mediate cell death, at later stages, GPR17 may even participate to repair mechanisms [14,15]. Thus, GPR17 may be proposed as a "sensor" of damage that is activated by the specific signaling molecules (uracil nucleotides and cysteinyl-LTs) that are released at high levels in the lesioned area, and as a new target for amyelinating post-injury responses. These data further highlight the attractivity of this receptor as a new target for drug discovery.

Recently, results obtained in recombinant systems, have been proposed GPR17 as a constitutive ligand-independent negative regulator of the CysLT1 receptor, that modulates CysLT1-mediated functions at the cell membrane [16]. Although this interesting hypothesis will have to be confirmed *in vivo*, it may be hypothesized that GPR17 may function as both a ligand-dependent and independent receptor depending upon specific patho-physiological conditions. Definitely, to fully understand the therapeutic potential of GPR17, specific ligands that do not interfere with the other P2Y or CysLT receptors are needed.

Along this line, as a first step to the design of selective ligands, we have recently provided a computational study of GPR17, providing a macroscopic view of a three-dimensional (3D) model of GPR17 complexed with three representative purinergic compounds: the endogenous agonist UDP and the synthetic antagonists MRS2179 and cangrelor [17]. To do so, we used a raw homology model of GPR17, based on the X-ray crystallographic 3D structure of bovine rhodopsin (*b*Rh) 1U19, deposited at RCSB Protein Data Bank http://www.pdb.org, the best high-resolution 3D template for a mammalian GPCRs that was then available [18]. In fact, for many years, the crystalline structure of the inactive receptor form of *b*Rh has been widely used as a template, even for significantly distant receptors, on the basis of the commonly accepted assumption that, in evolutionary related proteins, the 3D structure is more conserved than the amino acid sequence. Fortunately, within the GPCRs superfamily, one of the essential determinant for GPCRs activity concerns the 7TM architecture that is well conserved between all GPCRs.

Rhodopsin-based homology models of GPCRs and the subsequent structure-based drug discovery approach are widely accepted, since experimental data have indeed confirmed computational predictions for many GPCR models [19-21]. Recently, thanks to protein engineering, the modified structures of two human GPCRs have been solved, providing new templates suitable for homology modeling: the adenosine A_2*A *_receptor (A_2*A*_R) bound to the high-affinity antagonist ZM241385 (PDB code 3EML) [22]; the *β*_2_-adrenergic receptor-Fab (*β*_2_AR-Fab) (PDB code 2R4R) [23] and the *β*_2_-adrenergic receptor-T4 (*β*_2_AR-T4) (PDB code 2RH1) [24,25], both bound to their inverse agonist carazolol; the mutated *β*_2_-adrenergic receptor-(E122W)-T4 (*β*_2_AR(E122W)-T4) (PDB code 3D4S) bound to cholesterol and its partial inverse agonist timolol [26]. In addition, the crystal structure of the turkey *β*_1_-adrenergic receptor (*β*_1_AR, PDB code 2VT4), in complex with the high-affinity antagonist cyanopindolol, has been also solved [27], raising the issue of how a range of compounds with very different affinity values can bind to such closely related receptor subtypes. Finally, also the squid *Rh *(PDB code 2Z73) [28] has been determined. Analysis of the newly published crystal structures of the squid Rh, the human *β*_2_AR, the turkey *β*_1_AR and the A_2*A*_R further confirm that the TM7 core is conserved among the entire GPCR superfamily. Nevertheless, structural differences have been found even within the TM bundle. Comparison between *b*Rh and *β*_2_AR structures shows that the binding site of the ligand carazolol on *β*_2_AR is very similar to that of retinal on rhodopsin, despite the fact that carazolol is a diffusible ligand rather than a covalently-bound ligand like retinal. In contrast to the *β*-adrenergic ligands and retinal, the A_2*A*_R antagonist ZM241385, exhibits a significantly different orientation within the TM bundle. Interestingly, the bound A_2*A*_R ligand, while interacting with helices, gets in contact also with EL2 and EL3. The publication of such new structures allows a very detailed assessment on the reliability of models based only on ground state the *b*Rh: this is a unique GPCR, because of its light-induced activation mechanism driven by the *cis/trans *isomerization of its covalently-bound ligand. However, its structure has been solved with high resolution, in different crystallization environment, in different states and both with different methodologies (NMR and X-ray). In fact, a detailed analysis of the structure differences connected to crystal packing and binding states reveals that, in spite of the close similarity to the *b*Rh general architecture, mutual rearrangement of the helices involved in the activation mechanism are observed. Recently, also the crystal structure of the native retinal-free GPCR bovine opsin (*b*Ops) has been solved [29]: the breakage of the so-called ionic lock restraints the helical pack in the resting structure and allows a rotation along the axes of the helical bundle [30]. In our previous study, we utilized the rhodopsin-based model of GPR17, with or without ligands, and embedded in fully hydrated phospholipid bilayer. This model was then refined by means of docking combined with molecular mechanics (MM) and molecular dynamics techniques (MD). Our MD simulations on the rhodopsin-based model of GPR17 suggested that the primary nucleotide binding pocket in GPR17 is contained in an accessible crevice enclosed between transmembrane (TM) helices (mainly TM3, TM5, TM6 and TM7) and extracellular loop (EL) 2, in general agreement with the binding site proposed for small molecules to other class A rhodopsin-like GPCRs and for nucleotides to already known P2Y receptors [31-33]. Based on our computational data, we also hypothesized that at the extracellular interface of the receptor, the N-terminus (Nt) region and EL2 and EL3 form accessory binding surfaces that could address ligands to the deeper main binding pocket. We finally proposed that the driving force for binding of nucleotides to GPR17 was the electrostatic interaction between the phosphate groups of incoming ligand and the basic arginine residue at position 6.55 (See Ballesteros and Weinstein's numbering system for residues index [34]) that was a recurrent target for all the nucleotidic ligands docked in our GPR17 model. This residue belongs to the conserved motif H-X-X-R/K typical of all the related P2Y and CysLT receptors; this motif is commonly believed to be a key extracellular recognition for nucleotides since 1995, when the first hypothesis on nucleotides binding mode on P2Y_1 _was formulated [35-40]. The overall configuration of the identified binding pocket shares common features with the ones described for the P2Y receptors [33,41], albeit showing some interesting differences.

For the P2Y receptors, it's today commonly accepted that the driving force attracting nucleotides is provided by a triplet of conserved positively charged residues, buried in the TM bundle of the receptors: these are believed to interact with the negative charges of the phosphate groups of nucleotides [35-37]. For the P2Y_1_-subgroup, residues R3.29 (TM3), R/K6.55 (TM6) and R7.39 (TM7) have been proposed to be critical for nucleotide recognition. Between the three residues, only 6.55 is conserved as a basic one among all the P2Y receptor family members, and belongs to H-X-X-R/K motif cited above, whereas R3.29 and R7.39 are only typical of the P2Y_1_-subgroup. The last residue belongs to the Y-Q/K-X-X-R motif in TM7 and is shared by P2Y_1_, P2Y_2_, P2Y_4_, P2Y_6 _and P2Y_11 _[3,42]. In the P2Y_12_-subgroup, it was proposed that two lysines, one located in EL2 (immediately before the cysteine residue involved in the formation of the conserved disulphide bridge), and the other one located in TM7 at position 7.35 (belonging to the K-E-X-X-L motif conserved among P2Y_12_, P2Y_13_, and P2Y_14_), can account for cationic coordination of the phosphate moiety instead of residues R3.29 and R7.39. Furthermore, the residue close to the conserved cysteine in EL2 appears to be involved in interactions with the phosphates also for the the P2Y_1_-like receptors [33,41]. More recently, a variant of this model has been proposed for P2Y_14_, where two of the basic residues (6.55 and 7.35) are instead assumed to bind the hexose moiety of sugar-nucleotides [43]. Interestingly, multiple sequence alignment of GPR17 with P2Y family members showed that GPR17 lacks the basic triplet and the residue 6.55 binding the phosphates is the only one conserved in the putative pocket [17], despite the many positively charged amino acids typical of this peculiar receptor. We also found that the binding pocket appears to be shared by both nucleotide agonists and antagonists, even if the modality of binding differs in some details, highlighting a heterogeneity in the binding pocket recently arisen also within the P2Y receptor family.

Unfortunately, no definitive 3D model of any CysLT receptors complexed with their ligands has been proposed yet; so, a convincing hypothesis on the basis of recognition is currently unavailable, despite several anti-leukotriene agents are already available in the market and others have already successfully started their track in drug development trials. In this respect, the CysLT1 receptor antagonist zafirlukast (Accolate) was the first CysLT receptor antagonist to be marketed in the USA; montelukast (Singulair) has been introduced to market since 1998 in the treatment of asthma and allergic rhinitis [44]; pranlukast is still waiting for a global extension of its commercialization and it is currently available only in Japan [7,45,46].

In the present paper, to get more insight into the role of residues suggested to be crucial for the recognition mechanism by our previous computational data, the basic residue R6.55 of our GPR17 wild-type (WT) receptor model has been mutated to isoleucine, giving a mutant (R255I) receptor model of GPR17. The effects of this mutation on recognition nucleotides have been studied in silico, by simulating the "unbinding processes" of two docked ligands (the endogenous purinergic agonist the UDP and the leukotriene receptor antagonist pranlukast) from both the wild-type (WT) and the mutant (R255I) receptor model of GPR17 using steered MD (SMD) simulations. The comparison between the two simulations clearly shows that the energy required to force the unbinding of UDP from the WT receptor model was significantly higher than the work spent for the unbinding of the ligand from the R255I receptor. These data suggest that the same target residue (R255) could play a different role in either the recognition of distinct classes of ligands or in the modulation of receptor's activity when activated by ligands. Although the *in silico *hypothesis presented here still has to be confirmed experimentally, it represents an interesting starting point for *in vitro *validation. For example, according to our computational hypothesis, the actual involvement of the residue R6.55 in recognition of nucleotide phosphates has been also confirmed by experimental data recently produced by our group. Using a frontal affinity chromatographic-based method coupled to a mass spectrometry detection (FAC-MS), we evaluated the elution time of UDP and other nucleotide-derivative ligands on two chromatographic columns where cell membrane expressing both the native and the mutated form of GPR17 were entrapped on the surface of the stationary phase. For the natural agonist UDP, we found that the retention time on the WT receptor-containing column was higher than for the mutate receptor-containing column, suggesting that the lack of R255 may reduce the affinity for this ligand (unpublished data). In the present paper, we report the results obtained by applying the same computational approach to simulate the forced unbinding of the leukotriene receptor antagonist pranlukast, in order to investigate if the mutation affects the binding of pranlukast and if the putative target R255 is shared by the two molecules. At present, the study of the unbinding processes at atomic scale is available with the use of atomic force microscopy (AFM) [47], where external forces are applied to molecules to probe their mechanical resistance. The virtual mimics of such experiments are provided by steered MD (SMD) and constant force MD (CFMD), that mimic the so-called force-ramp and the force-clamp methods used in AFM, respectively. In the force-ramp method the mechanical resistance of biomolecules is measured applying a time-dependent force [48], while in the force-clamp methods a constant force is used [49]. With the use of such external forces, the MD path becomes irreversible and gives access to processes involving non-covalent bonds that cannot be achieved in the same time scale with the conventional MD simulations [50]. Based on SMD/CFMD, several reliable predictions of binding/unbinding [51-58] and folding/unfolding [59-65] processes have been obtained for various biological complexes. In unbinding experiments, the analysis of the interactions of dissociating ligands and the evolution of applied forces and ligand positions provide qualitative information about the irreversible work spent in the unbinding process: in this way, insights in structural features of receptor-ligand complexes and possible binding pathways are gained. However, our propose here was not to use SMD to define the exact ligand unbinding pathway/mechanisms, an issue that would require a more accurate analysis, but, as already mentioned before, to elucidate the role of R255 as possible target for GPR17 ligands. In fact, the simulations of the unbinding of ligands, such as UDP and pranlukast, from GPR17 receptor models presented here can also provide some attractive hypothesis on the unknown recognition mechanism and could thus be helpful to the planning of experimental mutagenesis studies and ligand affinity measurements.

## Results and Discussion

### Comparison between GPR17 and new templates

Our MD simulations study was performed on a *b*Rh-based homology model of GPR17 [17], for consistency with our previous study on GPR17, starting from a highly refined structure of the receptor. Nevertheless, recently, new GPCR structures have been solved and become available for comparative modeling (see Introduction). However, between the sequences of the currently available GPCR structures, for GPR17 the best alignment score was obtained with *b*Rh (19.3 for *b*Rh; 15.7 for human *β*_2_AR, 15.3 for turkey *β*_1_AR and 14.3 for human A_2*A*_R, Moe's alignment tool) that indeed still results as a good compromise for modeling GPR17 despite the lack on structural information on this receptor. To assure that the topology that we found for GPR17 was not an artefact due to the template, and also to assess if it was still reliable in view of the new GPCR structures, we compared our model with the structure of the human A_2*A*_R, the three structures of the human *β*_2_AR and the structure of the turkey *β*_1_AR. Both the structures of human A_2*A*_R, human *β*_2_AR and turkey *β*_1_AR showed the *α*-helical 7TM domain typical of the GPCR receptor family. Superimposition of the C-*α *atoms of the A_2*A*_R, the *β*_2_AR-Fab, the *β*_2_AR-T4, the *β*_2_AR(E122W)-T4 and the *β*_1_-AR to GPR17 and to *b*Rh is reported in Table 1. RMSD values obtained by rigidly superimposing the three structures to the GPR17 model vary from 1.955 to 2.867 Å, a range which is not significantly different from that obtained by superimposition of the same structures to *b*Rh.

**Table 1:** Alignment among GPR17 model and X-ray templates of GPCRs

RMSD (Å) after alignment of *α*-helical carbon					
	3EML	2R4R	2RH1	3D4S	2VT4

GPR17	2.867	2.458	2.688	2.738	2.413

*b*Rh	1.955	2.210	2.362	2.277	2.862

**Figure 1:**
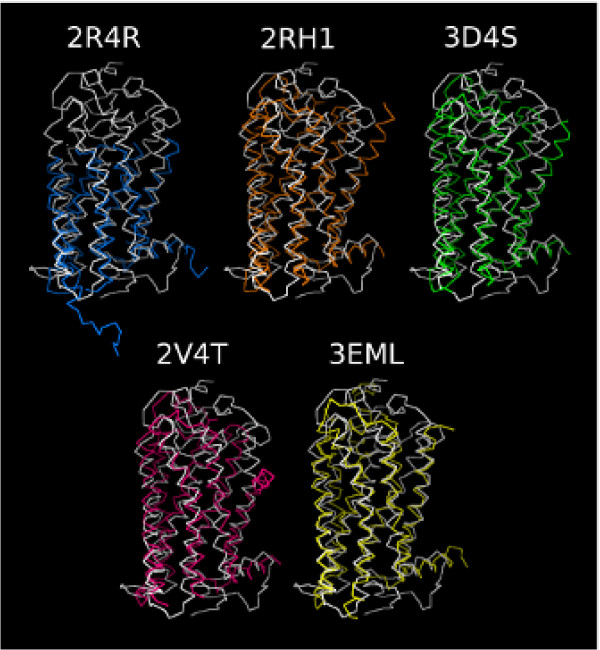
**Superimposition of *α*-helical domains of the *β*_2 _AR-Fab, *β*_2 _AR-T4, *β*_2_AR(E122W)-T4 *β*_1 _AR and A_2*A *_R structures to GPR17 model**. Ribbon representation of the *β*_2_AR-Fab (2R4R), *β*_2_AR-T4 (2RH1), *β*_2_AR(E122W)-T4 (3D4S), *β*_1_AR (2VT4) and A_2*A*_R structures after alignment of the *α*-helical domains to GPR17 model (in gray) are reported in cyan, orange, green, magenta and yellow respectively.

Globally, the helical pack was highly conserved among all the structures, and also the alignment of the *α*-helical domains to the GPR17 bundle yielded a good fit, as shown in Figure 1. In spite of the overall good fit among structures, there were some differences in the helical rearrangement concerning mainly TM1. In both *β*_2_AR and *β*_1_AR structures, this domain exhibited a kink corresponding to a hydrophobic motif (L-I-V-L-A-I-V) encompassing two helical turns that caused a marked outlying exposure of the N-terminus end of the helix: this hasn't been found either in our GPR17 model or in *b*Rh. Interestingly, all the available *β*-AR structures reveal the presence of an unexpected *α*-helix domain on the EL2, that is indeed significantly different from the *β*-hairpin organization that has been found for the EL2 of *b*Rh, suggesting that this feature could be a requirement for the binding of reversible ligands, and that a different accessibility to the binding pocket could exist among GPCRs [27]. Moreover, the A_2*A*_R structure reveals substantial differences in the architecture of the extracellular domains with respect to the other solved GPCR structures, as the EL2 is spatially constrained by two extra disulphide bridges that link this loop to EL1. At this time, we don't have any structural informations about the macroscopic arrangement of EL2 and of the other extracellular loops in GPCRs but, being the TM bundle so well conserved during evolution, it is reasonable to assume that at least some of the keys for selectivity reside in the extracellular region. This feature can also account for the exceptional plasticity of GPCRs and their capability to bind such a heterogeneous spectrum of molecules.

### Molecular dynamics of pranlukast

**Figure 2:**
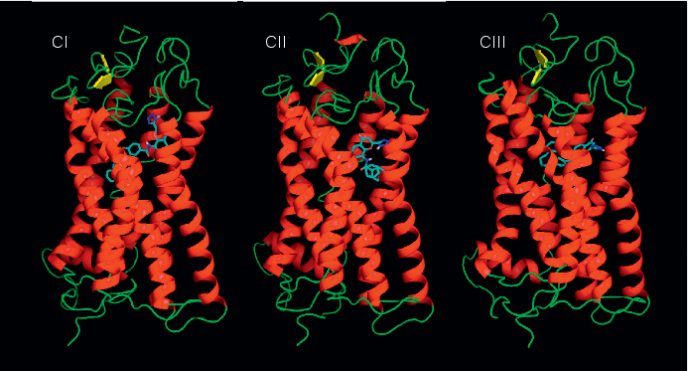
**Macroscopic view of three best configurations of pranlukast docked to GPR17**. The picture shows the three potential binding poses (CI, CII and CIII) obtained for the antagonist pranlukast (stick representation) on GPR17 (cartoon representation), by means of docking studies and 6 ns of molecular dynamic simulations. The chance of pranlukast to assume different and energetically comparable configurations, as for CI, CII and CIII, it is probably due to the high flexibility of the molecule yielding its high conformational freedom.

**Figure 3:**
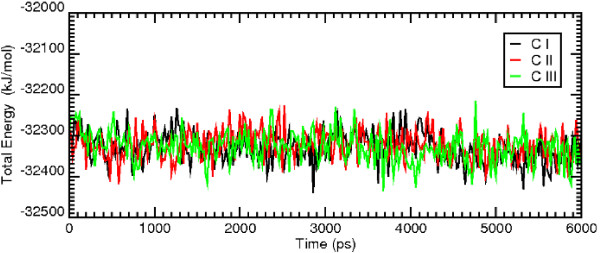
**Comparison of the energetic profile of the MD simulations of the three docking configurations pranlukast**. The plot shows the total energy profile as a function of time of the entire system membrane-receptor-ligand system during 6 ns of MD simulations, for the three different pranlukast configurations, here indicated as CI (in black), CII (in red) and CIII (in green).

**Figure 4:**
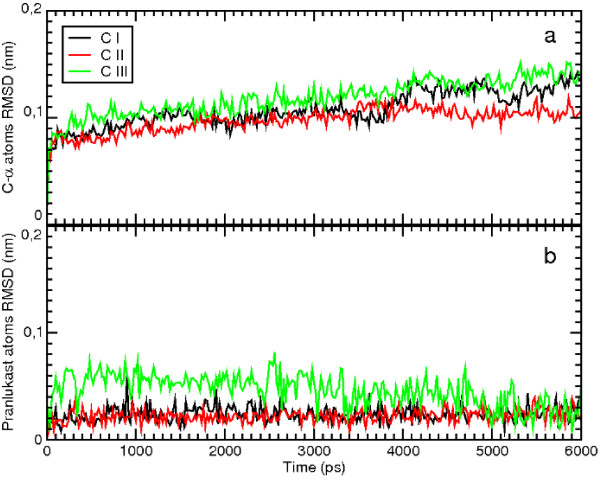
**Comparison of the RMSD of C-*α *and ligand atoms of the MD simulations of the three docking configurations of pranlukast**. The plot a shows the RMSD of C-*α *atoms of the protein as a function of time obtained for the MD runs of CI (in black), CII (in red) and CIII (in green). In panel b, the same colour are used to indicate the RMSD versus time of ligand atoms obtained for CI, CII and CIII simulations.

**Figure 5:**
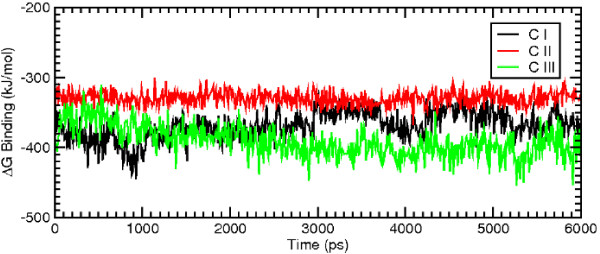
**Free energy estimate of the binding of pranlukast**. The free energy estimate of the binding of pranlukast for the 6 ns of MD simulations performed for the three different docking configurations is reported in black for CI, in red for CII and in green for CIII.

MD simulations of the WT model of GPR17 complexed with the receptor antagonist pranlukast have been performed, starting from the best docking configurations of pranlukast in its presumptive pockets. Docking studies, combined with MD simulations, unveiled three different potential binding sites for the antagonist pranlukast. In these pockets, pranlukast assumed three different configurations (CI, CII and CIII); all of them showed comparable docking energy and appeared realistic as hypothetical binding configurations to GPR17. Globally, these multiple binding modes are not surprising, because they account for the many degrees of freedom of pranlukast. The conformational analysis of the molecules give rise to many stable conformations which are very close in internal energy; hence the molecule can accommodate into the binding pocket with different features. These findings also suggest that the receptor can assume slightly different conformations and that the activation process may occur in a multi-step mechanism, where important conformational changes sequentially take place. The overall pictures of the antagonist-receptor complexes extracted from the 6 ns of MD runs are showed in Figure 2. We then computed the total energy of the system and the root mean square deviation (RMSD) for the MD trajectory of each of the three pranlukast configurations. Figure 3 and Figure 4a show, respectively, the energetic profile and the RMSD of C-*α *of the three pranlukast-GPR17 complexes, as a function of time. Both the total energy and the RMSD values of the C-*α *atoms of the protein are very close in value among the three different MD runs; moreover, after the initial relaxation, all of them keep a stable trend, at least for the last 2 ns of MD simulations, indicating that a relative stability has been reached. Figure 4b shows the RMSD values computed for pranlukast atoms in the three different MD trajectories. While the CI and CII conformations show a similar constant trend during the simulations, at the beginning of the MD run the CIII shows RMSD values higher than CI and CII. These values decrease to values similar to these of other two conformations only in the last ns of the simulation. Figure 5 shows the relative free energy difference (ΔG Binding) computed during MD simulations with the algorithm provided by the Gromacs analysis tool (see Methods for more details). Plots report an estimation of the relative ΔG Binding for the whole system, i.e. solvent-receptor-ligand. As the ligand is the same for all the three simulations, the reported values contain a constant additional factor, i.e. the energy corresponding to the free ligand in the solvent. Indeed, in our case, the subtraction of the energetic components of the runs of the free ligand in the solvent would not have affected the ΔG values and hence the interpretation of the data reported here. While apparently the most favourite trend was observed for CIII (lower ΔG Binding values) we wanted to focus our analysis on the conformation globally giving the most effective docking. Standard deviation for the three ΔG Binding profiles gave values of 21.28, 10.09 and 22.12 kJ/mol, for CI, CII and CIII, respectively. We noticed that, while displaying a higher energetic profile, pranlukast CII conformation was characterized by a more conserved and constant ΔG Binding with respect to CI and CIII. Moreover, it also displayed a smaller standard deviation: this was one of the reasons why we decided to perform our analysis on this pranlukast conformation. The binding energy computed by means of docking tools, both before and after the MD simulations varies from -13.21 to -14.93 kJ/mol for the three conformations; thus a selection based merely on the docking energy criteria is a bit risky, due to such a subtle difference. The previous observations, together with other evidence regarding the detail of the pose (see below), suggest that the binding mode is optimal and pranlukast is steadily docked into the pocket. A detailed view of the principal polar or hydrophobic interactions formed between pranlukast groups and residues within the pocket for CI CII and CIII is briefly presented below.

In CI, pranlukast assumes the more extended conformation with respect to CII and CIII, where, conversely, the molecules tended to fold into a closed conformation. In fact, in CI, pranlukast spanned the helical bundle, directing its tetrazole group toward the extracellular space close to TM6, TM7 and EL2, and extending its aromatic tail toward the inside region, parallel to the elongation axes of the protein. The main polar interactions formed by the tetrazole group within this conformation involved residues Thr175, Asn176 (EL2), Tyr251 (TM6) and Asn279 (TM7). The benzopyran and the 4-oxo oxygen atoms of the chromone region were hydrogen-bond (H-bond) acceptors for Tyr185 (EL2) and Arg255 respectively, whereas the oxygen of the phenylbutoxy-benzamide chain was H-bond acceptor for Arg87 (TM2). The phenyl chromone ring was surrounded by Tyr112 (TM3) and Tyr251 (TM6) at opposite sides, suggesting a possible formation of *π *- *π *interactions in both directions. Finally, the terminal phenyl established a few hydrophobic interactions with residues belonging to TM2 and TM5, such as Val81, Ile84 and Met115. In CII, the tetrazole ring lied close to R255 and H252, at a distance compatible with the formation of H-bonds. At present, we don't have any clear-cut information about the local protonation environment, but it is reasonable to believe that pH conditions could influence the acid-base equilibrium of the tetrazole function, that could indeed manifest its deprotonated state and express its acidic potential to form electrostatic interactions with the basic arginine and/or a protonated histidine. Other polar interactions concern the keto group of the chromone, that could act as H-bond acceptor for the hydroxyl groups of Tyr112 (TM3), Tyr251 (TM6) and Tyr185 (EL2). Phenyl portions of Tyr112 and Tyr251 can instead wrap the aromatic chromone portion of pranlukast forming typical *π *- *π *interactions. Aromatic portions of the residues Tyr116, Tyr120, Phe201, Phe205, Phe244, Phe248 enclosed both the terminal phenyl ring and the phenylbutoxy-benzamide group, giving rise to a big aromatic cluster, to which also hydrophobic residues such as Val249 and Ile119 take part. Moreover, cluster analysis applied to the MD trajectory, revealed that pranlukast within the CII configuration, despite its stability, underwent conformational changes and two different conformations were observed. In these two conformations the terminal phenyl group can alternatively share the *π*-electron cloud either with the phenyl ring of the phenylbutoxy-benzamide group, or with the phenyl ring of the Phe201 residue, forming an intramolecular or an intermolecular stacking interaction, respectively.

Also in CIII, pranlukast lied in a bent conformation, in which the chromone portion held a position approximately perpendicular to the protein *z *axis rather than parallel as in CII. Residues Gln183 (EL2), Tyr116 (TM3) Ser196 (TM5) and His252 (TM6) are potential H-bond donors/acceptors for tetrazole, whereas the benzamide carboxyl could form H-bonds with the backbone atoms of TM3 (Asn114-Tyr116). Within the presumptive pocket, aromatic and hydrophobic interactions were likely to be less defined than in CI and CII. An intramolecular stacking interaction between the terminal and the butoxy-benzamide phenyl was instead favoured, since the aromatic tail of pranlukast remained in a region of the protein close to the extracellular side and to TM1 and TM2, where hydrophilic amino acids are predominant.

In this respect, following the dynamical behavior of a few water molecules derived from the X-ray structure of *b*Rh and present in our GPR17 model, it has been previously proposed that TM3 can divide the helical bundle in two well distinct regions, each characterized by a different hydrophilic or hydrophobic profile [17]. TM4, TM5 and TM6 form a first hydrophobic region where highly conserved aromatic residues are predominant, with the exception of the region immediately below the interfaces with the extracellular space. Conversely, TM1, TM2 and TM7 determine a second hydrophilic region where all crystal water molecules tend to segregate, weaving a network of polar interactions with the surrounding residues that extends from the upper to the lower side of the protein: in this way a sort of "polar channel" is formed throughout the protein [17]. It would be interesting to clarify if this polar channel is an artefact of the model, or, alternatively takes part to signal transduction, as it has been demonstrated for the proton pump in bacteriorhodopsin (*b*R) [66,67], thus bridging extracellular events with the intracellular actors of the transduction machinary.

Unfortunately, we couldn't submit our results to a convincing critical comparison with previous independent data on other leukotriene-responding receptors, because only few models of such receptors and/or ligands have been developed so far, and the discussion on their reliability is still open. For example, several attempts for constructing a pharmacophoric model of CysLT1 receptor antagonists based on structure-activity relationships (SAR) studies have been reported, but they are not fully convincing, since none of them is in full accordance with the agreed features of the receptor. One of the difficulties in the development of a reliable 3D pharmacophoric model for CysLT1 antagonists resides in the flexibility of most antagonists. Furthermore, despite the fact that several antagonists share identical structural elements with the agonists (and a common binding site for agonists and antagonists has been proposed), the existence of structural overlapping between different classes of CysLT receptor antagonists and/or agonists is still debatable [68]. Based on quantitative SAR studies (QSAR), an hypothetical computational model of CysLT1 pocket has been built using almost rigid leukotriene antagonists as template; an arginine residue is incorporated into the preliminary model as an interaction site for the acidic moieties of antagonists [68]. This revealed additional interactions between the guanidine group and the nitrogen atoms of quinoline-containing CysLT1 antagonists. In some cases, the arginine residue could eventually interact also with *π*-clouds of phenyl moieties of CysLT1 antagonists. These data suggested that a pharmacophoric model based on structural similarity of agonists and antagonists may not be valid, and that antagonists do not necessarily bind to the same site or in the same manner as agonists. In the same model, the different alignment between pranlukast and montelukast suggested the presence of an additional pocket in the binding site for CysLT1 antagonists. Recently, a model of LTE_4 _complexed with P2Y_12 _receptors has been proposed, on the basis of *in silico *screening data, combined with intracellular calcium concentration measurements in CHO cells stably expressing a P2Y_12_-G16*α *fusion protein [11]. It has been demonstrated that, in addition to adenine nucleotides, the P2Y_12 _receptor, can respond to 5-phosphoribosyl 1-pyrophosphate (PRPP) and LTE_4_. For the LTE_4_-P2Y_12 _complex, it has been hypothesized that the 1-carboxylic acid and the 5-hydroxyl group of LTE_4 _interacted with Glu263 of TM6; the amino acid portion of cysteinyl group interacted with Leu284 of TM7, and the carboxylic acid with Ser101 of TM3. In conclusion, it is evident that additional advances need to be done in order to understand the cysteinyl-LTs binding modalities and to find a unifying theory that reconciles all the findings reported so far.

**Figure 6:**
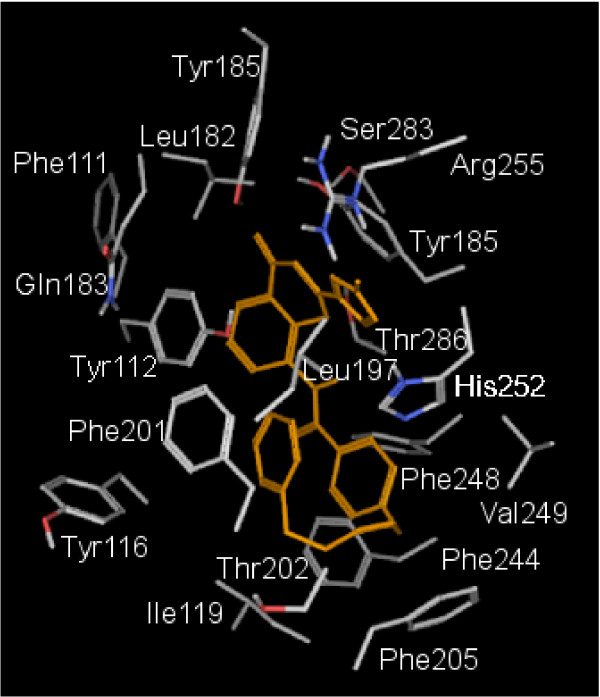
**Model of the pranlukast conformation CII**. Model of the complex formed by pranlukast and GPR17 after 6 ns of conventional MD simulation. Pranlukast is displayed in orange within the detailed binding pocket.

**Figure 7:**
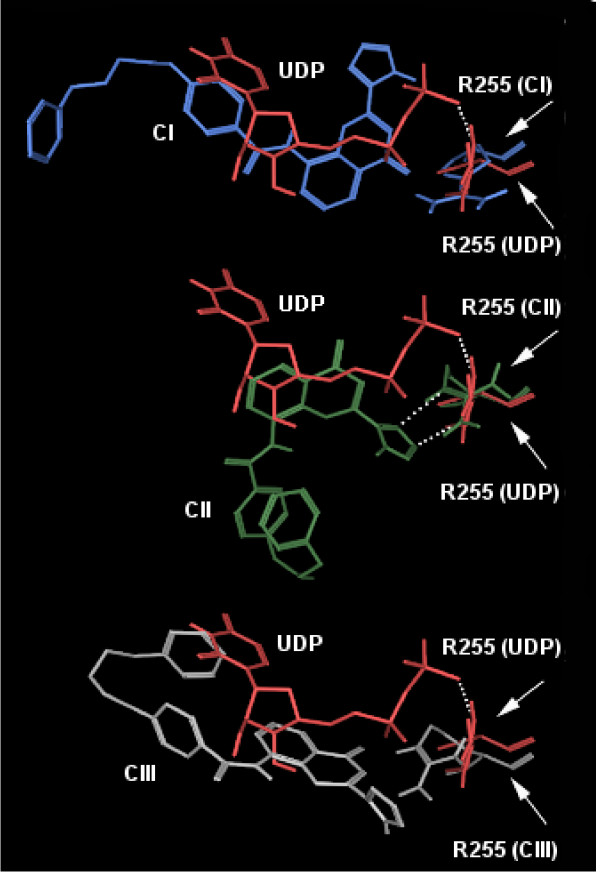
**Superimposition of UDP and pranlukast within the putative GPR17 binding pockets**. The picture shows the superimposition of UDP (in red) with the three docking poses of pranlukast (CI, in blue; CII, in green; CIII, in gray). For each simulation, the correspondent target residue R255, here highlighted with arrows, is reported in the same colors of either UDP or pranlukast. The hypothetical interactions between R255 and ligands are represented by white dotted lines.

On this basis, we focused our subsequent experiments on CII, where the tetrazole group of pranlukast is close to the H-X-X-R motif, and the phenyl rings are placed into a hydrophobic subpocket, that is highly conserved among GPCRs: this is in fact believed to be a common ancestral recognition target for this receptor superfamily [69]. Here, aromatic residues such as Phe201, Phe205, Phe244 and a triplet of embedded tyrosines belonging to TM3 held the aromatic portions of pranlukast. A detailed view of CII is showed in Figure 6. Several other reasons have led us to focus on this configuration. The first reason is that, according to this potential model, nucleotidic ligands and pranlukast would target the same arginine residue, providing an interesting issue to investigate. As cited before, an arginine residue has also been proposed as a partner for tetrazole or acidic functions of CysLT antagonists by Zwaagstra and coworkers, because of the favourable electrostatic potential of the guanidine group, of the ability to form multiple H-bonds in addition to the electrostatic ones, and finally because of the wide delocalization of the electronic-clouds of the guanidinium functional group. Figure 7 shows the superimposition of UDP with the three different poses of pranlukast within the putative GPR17 binding pockets identified by means of our docking and MD studies. Among the three docking poses obtained for pranlukast, only configuration CII (in green) is likely to share the target residue R255 with UDP (in red). In fact, while in the CII conformation pranlukast accommodates the tetrazole ring in proximity to the R255 residue, close to the phosphate chain of UDP (see dotted lines identifying the potential interactions), in the CI and CIII conformations, pranlukast is not likely to form interactions with the same residue. Moreover, the tetrazole group of pranlukast, that is directly connected to a rigid electron-rich system, recalls some structural elements of the 2-tetra-phenyl-containing subclass of the so called "priviledged structures". The latter are indeed selected scaffolds that are able to provide high-affinity ligands for more than one type of receptors, targeting common conserved motifs of the GPCR superfamily. Such structural motifs have been successfully used by many pharmaceutical companies to design "universal" pharmacophores and to synthesize combinatorial libraries, which are subsequently tested against novel GPCR targets, in an attempt to find lead compounds. In particular, for the 2-tetrazol-phenyl-containing subclass, it has been proposed that the phenyl moiety can be accommodated in a conserved aromatic TM subpocket formed by Phe6.44, Phe6.48 and Trp5.47 (TM5 and TM6), whereas the tetrazole ring lies close to residues positioned at positions 5.43, 6.52 and 5.42 or 6.55 [69]. In our GPR17-pranlukast CII complex model we observed a strong similarity with the interaction pattern predicted for priviledged structures: we therefore performed SMD study on this pranlukast configuration rather that on the other two. Finally, it is know that the key residue in the binding and/or activation mechanism of several GPCRs is a basic residue, frequently an arginine [70-75]: on this basis, we focused our attention on the Arg255 present on GPR17 that thus became an attractive target for the present study.

### Steered MD simulations

**Figure 8:**
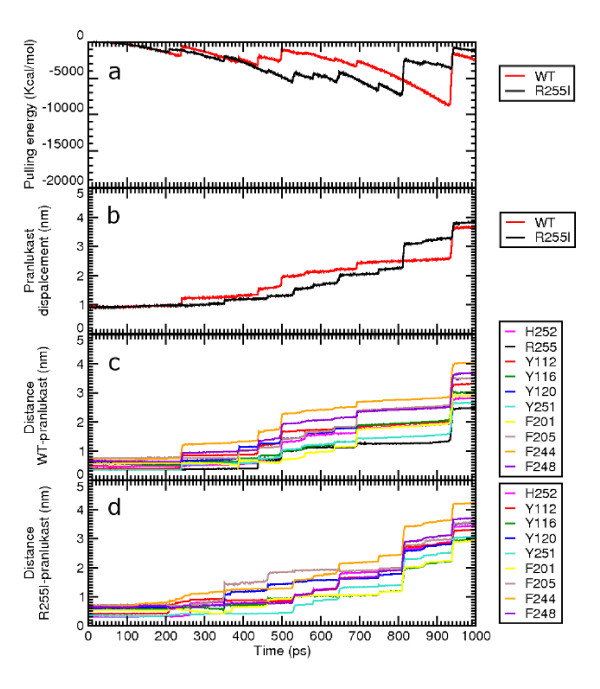
**Forced unbinding profile of pranlukast**. Panel a and b compare the unbinding simulations of pranlukast from the WT (in red) and the R255I (in black) receptor models: panel a shows the work developed to unbind pranlukast; panel b shows the displacement of the COM of the ligand from its starting position. Panel c and d show the distances between groups of atoms of the ligand that form polar or hydrophobic interactions with atoms of the WT or the R255I models, respectively.

**Figure 9:**
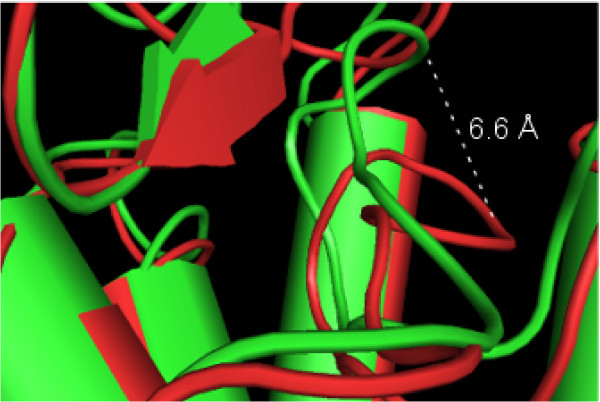
**EL2 dynamical behaviour**. Representative frames of the open and closed form of EL2, extracted from the SMD simulation, are reported in green and red, respectively. The picture shows a detailed view of the hinge movement of the loop that exhibits an extension up to 6.6 Å.

SMD experiments on GPR17-WT model, were set starting from the last minimized frame of the MD trajectory of the ligands UDP and pranlukast (CII). The residue Arg255 was then mutated to isoleucine, choosing the energetically favoured isoleucine rotamer among the ones proposed by the sequence mutation tool of the software Moe http://www.chemcomp.com. The R255I mutant model of GPR17 was thus obtained. Preliminary SMD experiments were performed choosing various pulling rates and pulling force entities, in order to validate the method on the two receptors. SMD experiments were then performed in parallel for both the WT and the R255I mutant model complexed with the ligands; then the mechanical resistance offered by the ligands through different unbinding pathways was measured. During the outgoing pathway from its pocket, the resistance yielded by the ligands was registered: both sterical factors and non-bonded interactions contributed to the observed peaks of force in the pulling steps. Here, for the interpretation of results, force peaks risen from SMD trajectory have been scaled to energetic values (kcal/mol) (see Methods for details). All the trial simulations provided comparable energetic profiles, although the elapsed time between similar events is different, as it depended on the pulling force/rate combinations. The analysis for the pulling experiments was focused on the results obtained using the following parameters: pulling rate  = 0.004 nm/ps and constant force *k *= 2000 kJ/mol nm^2 ^for a total duration of 1000 ps: this has proved to be the more equilibrate choice. As mentioned before, the SMD experiments were performed for both the WT and the R255I model on the CII pranlukast configuration. Here, the tetrazole group of pranlukast was close to position 6.55 on TM6, and the phenyl rings were embedded into the highly conserved aromatic/hydrophobic pocket enclosed among Phe201, Phe205, Phe244 and a triplet of tyrosines belonging to TM3. In Figure 8, the SMD unbinding trajectory of pranlukast from the WT and R255I receptors are compared. Panel a shows the pulling energy plot, computed for the WT (in red) and the R255I (in black) receptor models, respectively. No significant differences in terms of maximum value of energy were found comparing the two energetic profile for the two models; moreover, both energy profiles had values significantly lower than the ones observed for the unbinding of UDP from the WT receptor. This first observation on the energy involved in the unbinding suggests that the mutation of Arg255 did not definitely affect the binding of pranlukast to its binding pocket. The comparison of the SMD for the WT and the R255I receptor model (in panel a and b the WT and the R255I simulations are reported in red and black, respectively), shows only one relevant energetic peak in the case of R255I: this happened in correspondence with the transition of the ligand through the plug. This is highlighted also by the plot reported in panel b, where the displacement of pranlukast in the two simulations is reported. The pattern of the main interactions, computed as distances, between atoms of the pair of functional group involved in the bonds formation as a function of time, was similar for both the WT (panel c) and the R255I (panel b) receptor models. For the WT simulations, the interaction between Arg255 (in black) and tetrazole group persisted up to 440 ps, when also the interaction His252-tetrazole was broken (in magenta). Only a small peak corresponding to this event was found in the pulling energy plot for the WT simulation, as further confirmation that probably the mutation of Arg255 doesn't significantly influence the binding of pranlukast to GPR17. This suggests that the aromatic residues play instead a key role in the recognition of pranlukast. Several *π *- *π *interactions involving phenyl function of pranlukast and aromatic residues within the pocket were indeed observed for both the WT and the R255I receptor model, as shown in panel c and d, respectively. Among these, residues Tyr112, Tyr116, Tyr120, Tyr251, Phe201, Phe205, Phe244 and Phe244 are likely to accommodate pranlukast, thus representing a potential binding pocket for the antagonist. Moreover, analysis of the SMD trajectory for both WT and R255I receptors showed that, despite the constraints imposed by the conserved disulphide bond Cys104-Cys181 linking EL2 to TM3, during the traction of the ligands out of the receptor EL2 moved toward the extracellular space showing an hinge movement allowing the opening of the crevice on the top of the receptor. Measurement of the distance between the C-*α *atoms of the outmost residues in both the open and closed forms of EL2 yielded to a maximum span of 6.6 Å, as shown in Figure 9. For other GPCRs, this hinge movement, that highlights the very high flexibility of EL2, has been already associated with the activation mechanism, among which the 5-HT_4*A *_the complement factor 5a receptor C5a, the M3 and the related P2Y_6 _receptors [76-79].

## Conclusions

Here, we present a computational study of a *b*Rh-based homology model of the human GPR17 receptor, that extends our previous MD analysis of the purinergic component of this receptor and highlights some intriguing aspects of its dualistic nature. While this work was already in progress, the crystal structures of the first human GPCRs ad of additional receptors form other species have been published [22-28]. It was therefore critical to verify that the basic structural assumptions previously made by modeling GPR17 on *b*Rh were still true at the light of the new published structures. To do so, we superimposed the C-*α *atoms of the A_2*A*_R, the *β*_2_AR-Fab, the *β*_2_AR-T4, the *β*_2_AR(E122W)-T4 and the *β*_1_-AR to GPR17 and to *b*Rh.

The obtained RMSD values varied from 1.955 to 2.867 Å, a range which is not significantly different from that obtained by superimposition of the same structures to *b*Rh. On this basis, we conclude that the results presented here have general value and actually give information on the putative 3D structure of this new receptor. To further support the validity of our approach, in a recent publication, Costanzi was able to reproduce the docking pose of the ligand carazolol in two *b*Rh-based homology models of the *β*_2_AR. Comparison of the homology models with the X-ray structures of the β_2_AR elegantly demonstrated that realistic GPCR structures can be obtained through accurate modeling based on the *b*Rh structure [80]. Furthermore, *b*Rh-based homology models for other related receptors, i.e. the P2Y2 receptor, are still being proposed to exploit the most favourable sequence similarity with this template *b*Rh with respect to the sequence of the newly solved GPCRs [81]. This confirms that, in spite of their low sequence identity/similarity, all GPCRs share a common scaffold and supports the role of this approach as a powerful tool for the drug discovery process. Our data are also in line with some of the conclusions made for other GPCRs on the basis of these recently published crystal structures. For example, it has been reported that, in contrast to the *β-*adrenergic ligands and retinal, the A_2*A*_R antagonist ZM241385 exhibits a significantly different orientation within the TM bundle. Interestingly, the bound A_2*A*_R ligand, while interacting with helices, gets also in contact with EL2 and EL3. In a similar way, the involvement of EL2 in ligand binding to GPR17 was consistently predicted also by our SMD study, thus suggesting that this may represent a common characteristic of some specific GPCRs subgroups. This peculiarity adds diversity to the class A family of GPCRs and may play an important role in driving receptor selectivity.

Our specific challenge in the present study has been to use MD and SMD experiments as guide to the design of in silico site-directed mutagenesis experiments that, combined with ligand affinity measurements and hopefully further structural informations, could contribute to the design of new selective therapeutics for targeting GPR17. We focused our attention on Arg255, that has been proposed to play a crucial role in binding of other P2Y receptors to their nucleotide ligands [35-40], and substituted this Arg with Ile (R255I). Our SMD simulations, showing that the energy required to unbind pranlukast UDP was not significantly different between the WT receptor model and the mutated R255I, highlights the role of the basic residue Arg255 in the binding to nucleotides. No significant differences between the WT and the mutated receptor were instead found for the unbinding of the leukotrienic ligand pranlukast from GPR17; the magnitude of the forces used was also equal to the one used to unbind UDP from the R255I mutant receptor. Furthermore, pulling forces developed to break polar and aromatic interactions of pranlukast were comparable, suggesting that aromatic interactions are likely to play a predominant role in the recognition of pranlukast. Compared with our previous data obtained simulating the forced unbinding of UDP, the magnitude of the energy used to dissociate pranlukast form both the WT and the R255I receptor models was also near to the one used to unbind UDP from the R255I mutant receptor [82]. MD simulations thus suggest that the mutation of Arg255, while influences the binding of nucleotides to GPR17, does not affect the binding of pranlukast, indicating that two different subsites are present on GPR17 and that the intermolecular interaction networks with the ligands are different between UDP and pranlukast.

The existence of two different binding sites on this receptor, regardless of the agonist and antagonist nature of the ligands, is also consistent with the intrinsic difference in the chemical structure of the two classes of unrelated purinergic and leukotrienic ligands. Moreover, this hypothesis is also supported by the peculiar organization of the TM crevice, that, in GPR17, identifies two well defined areas with different hydrophilic/phobic surface profiles.

At present, the mechanism of activation and inactivation of the receptor is unknown, but some general hypothesis about the most probable target residues can be formulated, based on the present computational data. Regarding the putative nucleotide binding site, in GPR17, in agreement with the other members of the P2Y receptor family, the same binding cavity seems to be shared by purinergic agonists and antagonists, at least for small ligands. As described in our previous work [17], the antagonist cangrelor, due to its long aliphatic branches that depart from the nucleobase, can reach regions of the protein that are unaccessible to other nucleotide-derived ligands. Concerning the leukotrienic component of GPR17, the characterization of the binding site is even more uncertain, due to the flexible nature of the ligands for which the identification of the docked conformation to the CysLT1 and CysLT2 receptors has not been yet successful. Nevertheless, our data highlight the importance of the conserved aromatic/hydrophobic cluster for the recognition of pranlukast. Further investigations are needed to unveil whether this feature is shared by both agonist and antagonists. Finally, the hypothesis that two distinct binding sites, one for nucleotides and the other one leukotrienes, are present on GPR17 is in accordance with our previous experimental cross-antagonism data. It has been indeed demonstrated that, in 1321N1 cells heterologously expressing *h*GPR17, blockade of the cysteinyl-LT binding site with the CysLT antagonists montelukast or pranlukast did not abolish the response to uracil derivatives. In a similar way, blockade of the nucleotide binding site with either cangrelor or MRS2179 still permitted the response to LTD_4_. The computational approach presented here, with the support of other experimental strategies designed to confirm our hypothesis and to improve our computational model, could aid to elucidate, step by step, the molecular mechanisms at the basis of ligands-mediated GPR17 activation/inactivation.

With the present study, we also aimed at getting some hints on the overall mechanism of ligand recognition, i.e. not only on the role of the single amino acid residues, but also on the role played by the conformational rearrangement and mobility of protein domains, such as helices and loops. In fact, loop regions are currently deemed to be involved in the binding of large molecular weight ligands (i.e., peptides), while their role in the recognition of small molecules-responding GPCRs remains largely unresolved. It has been proposed that EL2 does not only provide a docking surface for the recognition mechanism, but could also act as a flexible "gatekeeper" in the binding of both allosteric and orthosteric GPCR ligands [83]. In agreement with this hypothesis, our SMD simulations unmasked the flexibility of EL2, that was not evident with conventional MD simulations run in the same time scale. Globally, these data advance our knowledge on the structure of the new hybrid receptor GPR17 and will eventually contribute to the design of "dual" ligands for this new target of high therapeutic relevance.

## Methods

### Preparation of the model of the membrane-GPR17-ligand system

A previously published rhodopsin-based homology model of the human GPR17 receptor embedded in a hydrated dipalmitoyl-phosphatidyl-choline (DPPC) bilayer and refined by means of MD was used as starting point for both the conventional MD and the SMD studies [17]. Docking studies and MD simulations of pranlukast were indeed performed on the same stable 3D structure of GPR17 coming from the 10 ns MD simulations and already used for the previous docking studies of GPR17 ligands. Locally minimized structure of the ligands docked into the membrane-receptor complex subjected to conventional MD simulations, were used as starting points for SMD experiments. The structure of the GPR17-UDP complex was taken form the already published model [17].

### Molecular dynamics simulations

The structure of GPR17 extracted from the 10 ns of MD simulation was submitted to a binding-cavity search using the Sitefinder tool included in the Delos package. Pranlukast was then docked into the suggested cavity using the docking tool included in the AutoDock 3.0 package [84], applying the genetic algorithm procedure to semiflexible docking module. AutoDock tools (ADT) were used to prepare the ligand and protein. A grid box with the dimensions 68 × 76 × 70 points for the *x*, *y *and *z *axis was constructed with grid points separated by 0.375 Å. The the population size was set to 50 and the rest of the parameters were taken as default according to the manufacturer's instructions. Among the docking configurations of proposed for pranlukast by AutoDock the three best energy scoring poses were chosen for further investigations by means of MD simulations. Pranlukast docked in the three configurations, together with the sidechains of the residues within 4.5 Å distance, was locally minimized, before starting MD run. Ligands topology for the MD runs were obtained from the automatic server PRODRG [85], using the standard Gromacs forcefield. The systems membrane-receptor-ligand was prepared for the MD simulations using a stepwise protocol. First, the systems were gradually minimized via the steepest descent allowing the various components to move individually with the following order: solvent and lipids, sidechains, the whole systems. The conjugate gradient method was then applied to improve the energy content of the system. The three ligand-receptor-membrane complexes were heated to the simulation temperature of 310 K in 300 ps and the three MD runs of 6 ns each were performed using the general conditions defined for this study and described in the "Computational details" subsection. The free energy calculations for the ligand-receptor complexes during MD were performed using a linear interaction energy (LIE)-based algorithm, as implemented in the MD software Gromacs. Using this method, the free energy is computed as an estimation of the difference in Gibbs free energy between two thermodynamic states of the system: the free energy of macromolecule with bound ligand in solution minus the free energy of the macromolecule in solution plus the free ligand in solution at standard concentrations, all at the same temperature and pressure [86]. In our case, for the comparison of MD simulations of pranlukast bound to GPR17, considering that our MD runs share the same ligand (and thus the same hypothetical simulation of the free ligand solution), we only computed the relative ΔG Binding between the three simulations of the complexes.

### Steered molecular dynamics simulations

To induce the unbinding of ligands from the receptor, an external force was applied to center of mass (COM) of the ligand, simulating retracting cantilever directed along an imposed vector (see below). Due to action-reaction principle, the spring acts as a sensor of all interacting processes along the selected exit pathway. The elastic force is proportional to the spring elongation relative to its equilibrium position, and it is given by the expression:  = *k*(*t *- ), where *t *-  is the displacement of the restrained atom with respect to its original position; *t *is time elapsed from the beginning of the simulation;  = 0.004 nm/ps corresponds to the velocity of the retracting cantilever and *k *= 2000 kJ/mol nm^2 ^is its force constant. In order to have a zero extra force at starting time, when the spring should be relaxed, displacement was set to 0 for time *t *= 0. For the interpretation of the results we converted the registered pulling force in energy values, by applying the following expression: *E *= (*t *- ). For the comparison of the unbinding processes of the agonist and the antagonists the mentioned combination for  and *k *was chosen among different values used in a previous series of simulations performed using combination of  values (0.001-0.01 nm/ps) and *k *values (2000-5000 kJ/mol nm^2^): the value of  was chosen so that the complete unbinding of the ligands occurs within 1 ns for each SMD simulation, that seemed to us a good compromise between in saving computational time and register atomistic events. The value of *k *was chosen to obtain a stiff spring in a drift regime. The vector along which ligands were pulled apart was imposed for both ligands parallel to the *z *axis of the protein, and also parallel to the principal axis of the membrane that, for a GPCR, is likely to correspond to the only exit path possible for a bound ligand. To define the exact values of the components (versors) of the pulling vector, an hypothetical end point of the unbinding pathway for both ligands was chosen by superimposition of the two ligands in the extracellular solvated environment. Then, by means of a homemade script, we computed the values of the three versors defining the direction of the hypothetical vector along the *z *axis and connecting the COM of each ligand in the starting position and the COM of the ligand in the final position. To ensure that velocity kept a constant value for the whole simulation, a combination of the *x*, *y *and *z *versors was then computed in a such way that they would give a unit vector.

### Computational details

All the simulations were run on a Linux cluster Blade with Xeon processors. All the minimization steps, MD, SMD simulations and concerning analysis were curried out using the Gromacs 3.3 package [86,87]. All the MD and SMD runs were performed using the Gromacs force field, modified by all the parameters necessary for the description of each component and their reciprocal interactions, based on manifacturer's instructions; the periodic boundary conditions were applied in all three *x*, *y *and *z *dimensions. The isothermal isobaric NPT ensemble (constant number of particles, pressure and temperature) was applied. Solvent (water molecules and chloride ions) and non-solvent (lipids, protein and ligands) component of the system was separately coupled to a temperature bath at 310 K, with a coupling constant τ_*t *_of 0.1 ps. The pressure coupling was set as independent in the *x *and *y *directions (semi isotropic coupling), with a constant pressure of 1 bar and a coupling constant τ_*p *_of 1 ps. A 2 fs time step was used for the integration of the equations of motions and all bond distances involving hydrogen atoms were constrained using LINCS [88]. Configurations were saved for every 1 ps for analysis. The analysis of the trajectories were computed with the specific Gromacs tools.

## Authors' contributions

CP carried out the computational study, performed the analysis of the data and drafted the manuscript, GR participated in the drafting and in the design of the study, MPA conceived of the study, provided the biological background for data interpretation and helped to draft, PF participated in its design and coordination. All authors read and approved the final manuscript.
